# Usefulness and Limitations of Multiple Ligation-Dependent Probe Amplification in Antithrombin Deficiency

**DOI:** 10.3390/ijms24055023

**Published:** 2023-03-06

**Authors:** Rosa Cifuentes, José Padilla, María Eugenia de la Morena-Barrio, Belén de la Morena-Barrio, Carlos Bravo-Pérez, Pedro Garrido-Rodríguez, María Llamas, Antonia Miñano, Vicente Vicente, María Luisa Lozano, Javier Corral

**Affiliations:** Servicio de Hematología y Oncología Médica, Hospital Universitario Morales Meseguer, Centro Regional de Hemodonación, Universidad de Murcia, IMIB-Pascual Parrilla, CIBERER, 30008 Murcia, Spain

**Keywords:** antithrombin deficiency, multiplex ligation-dependent probe amplification, structural variants, genetic variants

## Abstract

Multiplex ligation-dependent probe amplification (MLPA) identifies genetic structural variants in *SERPINC1* in 5% of cases with antithrombin deficiency (ATD), the most severe congenital thrombophilia. Our aim was to unravel the utility and limitations of MLPA in a large cohort of unrelated patients with ATD (N = 341). MLPA identified 22 structural variants (SVs) causing ATD (6.5%). MLPA did not detect SVs affecting introns (four cases), and the diagnosis was inaccurate in two cases according to long-range PCR or nanopore sequencing. MLPA was used to detect possible hidden SVs in 61 cases with type I deficiency with single nucleotide variations (SNVs) or small insertion/deletion (INDEL). One case had a false deletion of exon 7, as the 29-bp deletion affected an MLPA probe. We evaluated 32 variants affecting MLPA probes: 27 SNVs and 5 small INDELs. In three cases, MLPA gave false-positive results, all diagnosed as deletions of the affected exon: a small INDEL complex, and two SNVs affecting MLPA probes. Our study confirms the utility of MLPA to detect SVs in ATD, but also shows some limitations in detecting intronic SVs. MLPA renders imprecise and false-positive results for genetic defects which affect MLPA probes. Our results encourage the validation of MLPA results.

## 1. Introduction

Structural variants (SVs) include a large and heterogeneous group of genetic alterations of more than 50 bp, including gross genetic variations of key pathogenic relevance such as deletions, duplications, insertions, inversions or chromosomal translocations. Although they are less frequent in the human genome than single nucleotide variants (SNVs) or small insertions or deletions (INDELs), they involve a larger number of base pairs and, therefore, have a greater pathological impact [1].

The methods used to detect SVs are different from those used to identify SNVs and INDELs. In fact, current sequencing methods based on short reads, which effectively identify SNVs and INDELs, may fail to detect and characterize SVs. Multiplex ligation-dependent probe amplification (MLPA), a targeted analysis multiplex PCR technique, has been widely used to detect SVs in many genetic disorders [2]. This method only detects gain or loss of genetic material (usually exons), but does not accurately report SV length and does not achieve nucleotide resolution [2].

Congenital antithrombin deficiency is the most severe congenital thrombophilia, as this serpin is the main endogenous anticoagulant that effectively inhibits multiple procoagulant proteases, mainly thrombin and FXa. Thus, a functional defect in a single allele of *SERPINC1*, the gene encoding antithrombin, by a dominant mechanism, significantly increases the risk of thrombosis. Moderate antithrombin deficiency (80–90% of values observed in a reference plasma) only mildly increase the risk of thrombosis [3]; mutations abolishing the function of the affected allele (50% of values observed in a reference plasma) increase the risk of thrombosis up to 50-fold, and complete antithrombin deficiency (0% of values observed in a reference plasma) results in embryonic lethality [4]. Two different types of antithrombin deficiency have been described in patients with congenital deficiency [4]: type I, if the gene defect causes null or severely impaired transcription, translation, or secretion of the variant protein. Patients carrying type I deficiency have reduced activity and antigen levels in plasma; type II, if the gene defect impairs or abolishes the anticoagulant activity of the variant molecule which is secreted. Patients carrying type II deficiency have reduced activity but nearly normal antigen levels in plasma. The high risk of thrombosis associated to antithrombin deficiency and the benefits of antithrombin deficiency treatment in carrier management including, in addition to conventional anticoagulants, the use of antithrombin concentrates, are data that strongly support the correct diagnosis of an antithrombin deficiency in patients with thrombophilia [5].

In *SERPINC1*, from the available data of the Human Gene Mutation Database (HGMD), 90.4% most of the defects causing antithrombin deficiency are SNVs and INDELs *SERPINC1*. Therefore, sequencing of the seven exons and flanking regions of this gene is the first strategy used to identify the molecular basis of this thrombophilia [4,6]. Interestingly, about 5% of cases are explained by SVs, which are detected by MLPA targeting the seven exons of this gene [4,6,7]. However, the proportion of SVs causing antithrombin deficiency may be underestimated, as SVs affecting introns, such as deletions or insertions of retrotransposons, are not detected by MLPA [8,9] and some SVs affecting exons, such as exon 6 duplication, can be barely detected by MLPA [10]. Moreover, the high proportion of repetitive elements, mainly Alu sequences, in and around SERPINC1 [11], together with the relevance that these elements play in the generation of SVs affecting this gene and causing antithrombin deficiency [12], encouraged us to carry out a comprehensive analysis of the involvement of SVs in antithrombin deficiency.

In this study, we analyzed the usefulness and limitations of MLPA in antithrombin deficiency, focusing on the search for potential SVs that might be obscured by the current algorithm designed to characterize the molecular basis of this severe thrombophilia, which restricts the analysis of potential SVs by MLPA only to cases with type I deficiency and no pathogenic genetic variants identified by the sequencing of *SERPINC1*. The results obtained confirmed the usefulness of this method to detect SVs affecting *SERPINC1* exons but also showed some limitations of MLPA that should be taken into account for this and potentially any other genetic disease using this molecular method.

## 2. Results

### 2.1. Antithrombin Deficiency-Causing SVs Detected by MLPA

**Figure 1:**
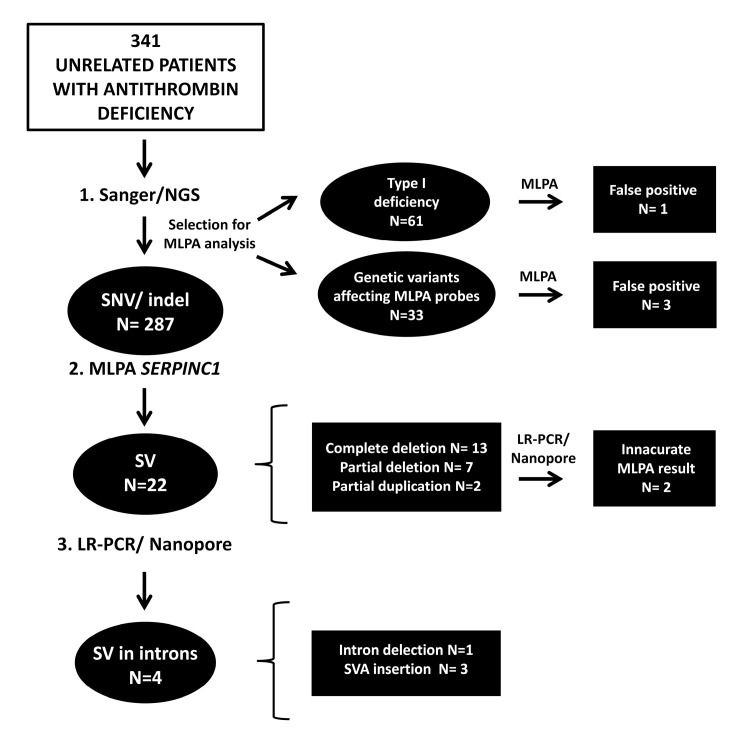
Genetic defects identified in our cohort of patients with confirmed antithrombin deficiency following the current diagnostic algorithm for molecular characterization of this disorder. Also shown are cases selected for screening of SVs by MLPA because they are carrying: (i) an SNV or INDEL and had a type I deficiency or (ii) a genetic variant in areas potentially sensitive to MLPA probe binding.

The molecular algorithm used in our laboratory, described in detailed elsewhere [13,14], follows the indications of the International Society of Thrombosis and Haemostasis (ISTH) to identify the genetic defects causing antithrombin deficiency [6]. This procedure first screened SNVs and INDELs by Sanger sequencing of the seven exons and flanking regions of *SERPINC1*. This approach yielded very efficient results, as 284 cases from our cohort carried 105 different *SERPINC1* defects potentially causing the deficiency. Next, the entire *SERPINC1* gene was sequenced in the cases with negative results finding three mutations with regulatory effect in the promoter or intronic regions. MLPA analysis was restricted to screening for SVs in the 54 cases with negative results after the previous sequencing approaches. This method identified 22 SVs causing antithrombin deficiency in our cohort, mainly deletions. Thirteen cases had complete *SERPINC1* deletions, seven partial deletions and two partial duplications. In the remaining 32 cases, whole-gene analysis by long-range PCR and NGS sequencing and long-read whole-genome sequencing using nanopore identified two different SVs in four cases with previously unknown molecular basis using the former techniques: three unrelated cases carried insertions of a retrotransposon in intron 6 [12], whereas in the remaining patient, a deletion covering most of intron 1 was detected [8]. The segregation of these genetic defects and antithrombin deficiency in family studies was confirmed. The algorithm followed to identify the molecular basis of antithrombin deficiency in our cohort and the *SERPINC1* gene defects found, including the SVs identified, are shown in Figure 1.

### 2.2. Analysis of SVs by Alternative Techniques to MLPA

The nucleotide characterization of the SVs identified in our cohort, performed by LR-PCR and NGS and/or nanopore sequencing showed six cases in which the MLPA diagnosis was either not accurate (N = 2) or in which the SVs were not detected (N = 4) (Figure 1).

**Figure 2:**
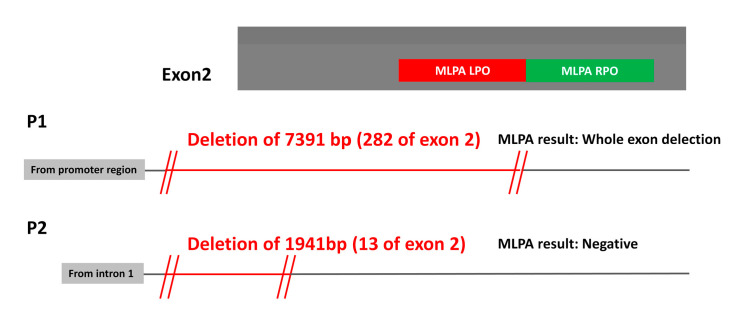
Representation of two *SERPINC1* deletions affecting exon 2 not precisely identified by MLPA in two patients with antithrombin deficiency. The length of the deletion, the position of the MLPA probes and the results generated by this technique are indicated.

The first case (P1) had a heterozygous deletion of exons 1 and 2 according to MLPA. However, LR-PCR revealed a 7391 bp deletion spanning exon 1, intron 1 and 282 bp of exon 2 (i.e., a partial deletion of exon 2) but involving one MLPA probe and part of the second probe (Figure 2). This finding explains why MLPA wrongly diagnosed a complete deletion of exon 2 in P1. The second case (P2) presented the opposite scenario. LR-PCR revealed a 1941 bp deletion affecting the 3’ end of intron 1 and 13 bp of exon 2 [8] without affecting the MLPA probes (Figure 2). Thus, MLPA analysis of this case did not detect any deletion.

In the third case (P3), the exon 1 deletion detected by MLPA was indeed a complex SV that also involved exons 2 and 3 [9].

In addition, the retrotransposon insertion in intron 6 identified in three cases (P4–P6) was not detected by MLPA [12].

### 2.3. Search for Hidden SVs

Because current molecular diagnostic algorithms terminate once a potential causative defect is detected in *SERPINC1* after sequencing, SVs are not evaluated in cases with a pathogenic or probably pathogenic SNV or INDEL identified by sequencing methods, particularly if segregation analysis in family studies supports a link between the *SERPINC1* mutation and antithrombin deficiency [4,6]. This raised the concern that SVs might not be identified if they occurred in a patient carrying an SNV or INDEL. We speculated that this situation might be more plausible in cases carrying genetic defects with low prediction of pathogenicity or associated with an unexplained phenotype (such as missense mutations causing type I deficiency). Moreover, we considered that partial deletions or duplications are the SVs most likely to be found in these patients, as complete deletions might result in apparent homozygosis, which has been never found except in some type II deficiencies. To clarify this concern, we selected 61 cases with a positive finding in *SERPINC1* obtained by sequencing methods that had type I deficiency, and performed MLPA analysis to evaluate possible hidden SVs (Figure 1, and Supplementary Table S1).

**Figure 3:**
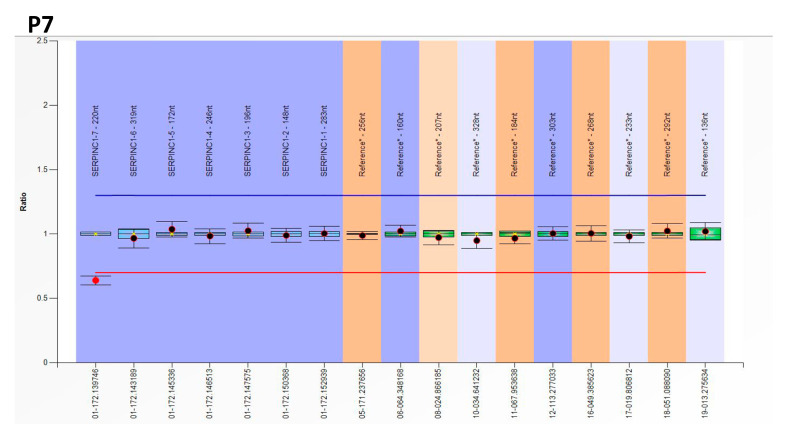
MLPA identification of a heterozygous deletion of exon 7 in patient P7 with antithrombin deficiency who carried a genetic defect previously identified by sequencing all 7 exons and flanking regions of *SERPINC1*. Blue line shows the cut-off to consider gain of genetic material (duplication) and the red line loss (deletion).

Only one case (P7) had a heterozygous deletion of exon 7 according to MLPA results (Figure 3).

**Figure 4:**
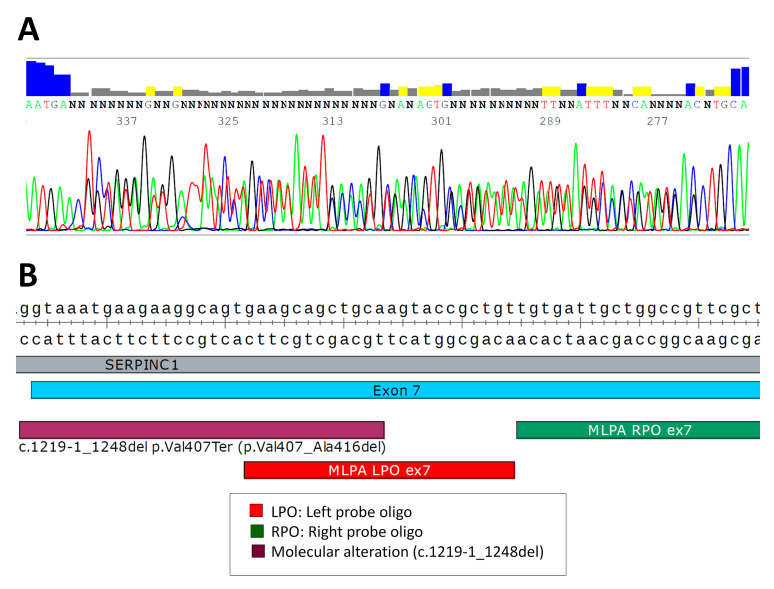
Genetic defect identified in patient P7 by Sanger sequencing: (**A**) electropherogram of the frameshift caused by the 29 bp deletion; (**B**) schematic representation of the location of this small deletion in the *SERPINC1* gene. The location of the MLPA probes covering exon 7 (blue) and the small deletionare also shown.

However, Sanger sequencing of the 7 exons and flanking regions of P7 revealed a 29-bp heterozygous deletion covering the last nucleotide of intron 6 and the first 28 nucleotides of exon 7: c.1219-1_1248del. This defect affected the correct splicing of this exon, and this is probably its pathogenic mechanism. However, this small deletion also partially affected the MLPA-LPO probe (Figure 4), which explained the inaccurate result diagnosis of whole exon 7 deletion observed with MLPA.

### 2.4. MLPA False Positives

The results obtained in patients P1 and P7 suggested that small genetic defects affecting the binding of MLPA probes may be misinterpreted as whole exon deletions when using this method. To test this hypothesis and characterize the defect that could cause incorrect diagnosis by MLPA, we analyzed by MLPA 32 additional patients with different *SERPINC1* genetic variants close to the binding sites of the probes used in MLPA in different exons: 27 SNVs, 3 small deletions, 1 small insertion, and 1 small complex variant (Figure 1 and Supplementary Table S2).

**Figure 5:**
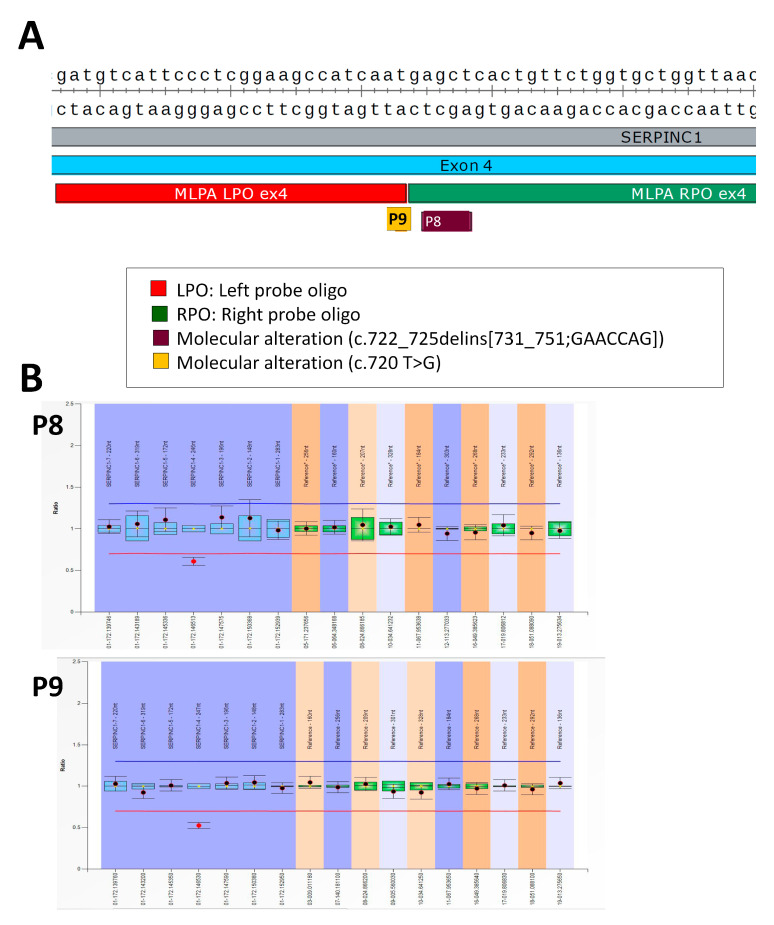
Genetic defects identified in patients P8 and P9: (**A**) localization of the small complex INDEL identified in P8 and the SNV of P9; and positions of MLPA probes covering exon 4 of *SERPINC1*; (**B**) MLPA results obtained in P8 and P9. In both cases, MLPA incorrectly identified a heterozygous deletion of exon 4.

In three cases, MLPA mistakenly identified a deletion of the entire exon affected by the genetic variant. The first case (P8) had a small complex INDEL complex in exon 4 c.722-725delins[731_751:GAACCAG] causing a 28-bp duplication and a 4-bp deletion that surprisingly caused a peculiar type II deficiency [15]. MLPA identified P8 as carrying a complete deletion of exon 4 (Figure 5).

The second case (P9) had an SNV: c.720 T > G affecting the last nucleotide of the MLPA LPO probe of exon 4. MLPA also characterized P9 as carrying a complete deletion of exon 4 (Figure 5).

**Figure 6:**
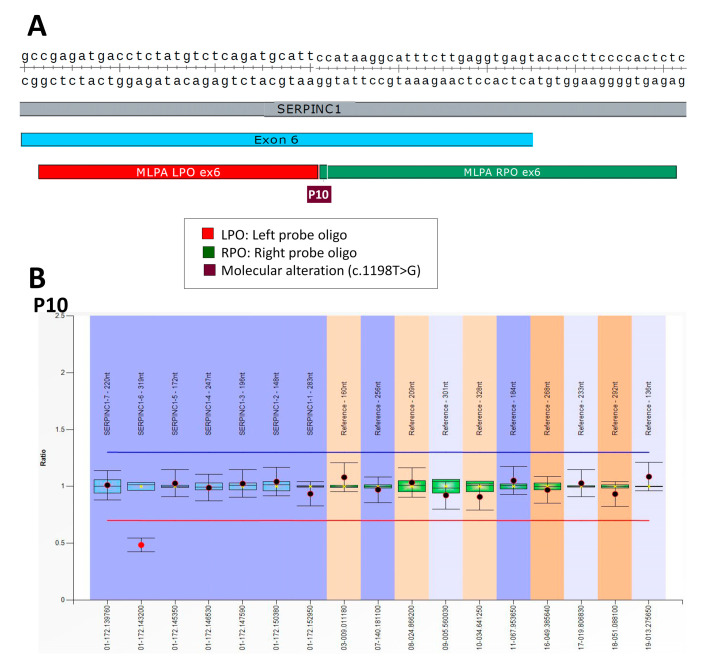
Genetic defect identified in patient P10: (**A**) localization of SNV and MLPA probes in the structure of exon 6 of *SERPINC1* (blue); (**B**) MLPA result obtained in P10 that incorrectly recognized a heterozygous deletion of exon 6.

The last case (P10) was, like P9, a patient carrying an SNV located in exon 6 (c.1198T > G) affecting the last nucleotide of the MLPA LPO probe of this exon. This genetic defect was also characterized by MLPA as a heterozygous deletion of exon 6 (Figure 6).

## 3. Discussion

Variations in the human genome are responsible for the diversity of human beings, but they also affect the susceptibility to genetic diseases [16]. The correction of these pathogenic genetic variants is the ultimate goal of gene therapies that aim to definitively cure the causative disorder [17], so the correct characterization of the real pathogenic gene defect causative of a disorder in any patient is crucial. Mass sequencing methods (NGS) have revolutionized the screening of genetic defects and have contributed to the development of modern molecular medicine [18]. These methods accurately detect SNVs. The main challenge regarding the finding of SNVs in patients by NGS methods is to accurately predict or determine their functional and pathological consequences [19]. In contrast, SVs which likely have more pathological impact than SNVs or INDELs, unfortunately also have a more complex diagnosis, as they may be lost by NGS methods, and usually they cannot be fully characterized by other molecular methods such as FISH, CGHa, SNPa, or MLPA [1,2]. Therefore, it is of interest to improve the identification and characterization of SVs, particularly those involved in diseases. In this study, we have evaluated the strengths and limitations of one method widely used to detect SVs in clinical practice: MLPA. We performed it in a key genetic disorder: antithrombin deficiency, by evaluating a large cohort of patients with this rare disease. The conclusions obtained for this thrombophilia can be extrapolated to any other disorder also using this methodology for diagnosis.

Our study confirms the percentage of cases with antithrombin deficiency explained by SVs: 5–8% [7]. Furthermore, we explored, for the first time, the possibility that the current diagnostic algorithm for studying the molecular basis of antithrombin deficiency [6], which ends in the search for further genetic defects when an SNV or INDEL is detected, may miss pathogenic SVs. This potential misdiagnosis of SVs causing antithrombin deficiency could be of great importance in two different frameworks: (i) from a therapeutic point of view, if considering a potential gene therapy. The isolated identification of an SNV without the diagnosis of linked and missed SVs might lead to designing gene therapy approaches targeting the SNV that would never cure the disorder. The accurate curative gene therapy approach for these cases, as for any carrying causative SVs, would be adenoviral vector transfer of antithrombin; (ii) from a diagnostic and prognostic perspective, the identification of an SV impairs the prognosis compared with the identification of an SNV, as usually SVs are associated with a worse prognosis and higher pathogenicity than an SNV.

However, screening for SVs by MLPA in 61 candidate cases, all patients with antithrombin type I deficiency carrying SNVs or small INDELs, revealed that none carried SVs. This result suggests that, if present, the combination of a mutation SNV or INDEL and an SV is very rare in antithrombin deficiency. Furthermore, this result confirms the high sensitivity, both genetic as well as conformational and functional, of antithrombin to even minor modifications, making most genetic defects affecting *SERPINC1*, including SNVs, potentially pathogenic. In fact, no missense polymorphism with an allele frequency < 10^−3^ has been described in *SERPINC1* and only 11 missense variants with an allele frequency > 10^−4^ have been identified in this gene, most of them with demonstrated pathogenic consequences (https://gnomad.broadinstitute.org/gene/ENSG00000117601?dataset=gnomad_r2_1 Last accessed 30 September 2022).

Interestingly, this study also shows some limitations of MLPA for diagnosing antithrombin deficiency, which may be extrapolated to any other disorder, which deserve further attention:

MLPA detects all SVs affecting *SERPINC1* exons regardless of their type (deletion or duplication) [12], but this method does not detect SVs affecting introns of this gene, such as deletions [8], or retrotransposon insertions [9], despite the fact that these defects also cause antithrombin deficiency. The design of MLPA intron probes to test for these defects should be considered, since they affect about 1% of cases with antithrombin deficiency. In addition, our study also confirms the limitations of MLPA in identifying certain genetic defects affecting MLPA probes [20]. Thus, whereas deletions or duplications affecting the whole exon are detected, small insertions, deletions, or even SNVs can give false or inaccurate results depending on the location of the defect in the MLPA probes. Thus, if the deletion does not cover the probe, it is not detected and a false-negative result can be reported. Conversely, if the gene defect affects a high number of nucleotides of the probe or the genetic defect is located in the junction of both probes, even with minor consequences in the rest of the exon, such as SNVs, MLPA could diagnose these defects as a complete exon deletion. We have given five examples of false positives generated by MLPA in antithrombin deficiency. It is important to remark that only SNVs or INDELs affecting the junction of the MLPA probes, but not any other SNVs or INDELs, cause false positive MLPA results. These results should encourage: (1) a careful design of MLPA probes to rule out single nucleotide polymorphisms affecting the boundary region, and (2) the requirement for validation of MLPA results to rule out potential artifacts. Nanopore sequencing with adaptive sampling may be a good approach for this validation [15].

## 4. Materials and Methods

### 4.1. Patients

The study was performed on a cohort of 341 unrelated Caucasian cases from different hospitals with confirmed antithrombin deficiency, identified during a 24-year enrollment period (1998–2022). All patients gave informed consent following ethical guidelines, blood samples were collected, and plasma and DNA was obtained.

### 4.2. Characterization of Antithrombin Deficiency

Characterization of antithrombin deficiency included functional assays by chromogenic methods (Anti-FXa and Anti-FIIa), antigen quantification by home ELISA or immunodiffusion, evaluation of plasma antithrombin by Western blot using different electrophoretic conditions (denaturing and native, the latter with and without 5 M urea, to identify the latent conformation), and identification and semi-quantification of forms with low affinity to heparin by cross-linked immunoelectrophoresis.

### 4.3. Molecular Analysis

Genomic DNA was purified from peripheral blood nucleated cells by standard procedures.

Genetic variants of *SERPINC1* were determined by sequencing the 7 exons and flanking regions, as well as the promoter, using primers and conditions described previously [13]. Complete sequencing of *SERPINC1* was performed in cases with negative results, following a high-throughput sequencing (NGS) procedure [14].

SVs were evaluated by MLPA using the SALSA MLPA Kit P227 *SERPINC1* (MRC Holland, Amsterdam, The Netherlands).

Characterization of SVs at the nucleotide resolution level was performed in samples with available DNA by two methods: (i) long-range PCR (Long Amp Taq NEB) and Sanger sequencing (Myseq, Illumina, San Diego, CA, USA), or (ii) nanopore sequencing, either by enrichment of a region containing *SERPINC1* by adaptive sampling on a MinION device (ONT, Oxford, UK), or by whole-genome long-read sequencing on a PromethION device [12].

## 5. Conclusions

Our study confirms the utility of MLPA to detect SVs in ATD, but also shows some limitations in detecting intronic SVs. MLPA renders imprecise and false-positive results for genetic defects which affect MLPA probes. Our results encourage the validation of MLPA results by other methods.

## Data Availability

Data is available upon request to the corresponding author.

## Supplementary Materials

The following supporting information can be downloaded at: https://www.mdpi.com/article/10.3390/ijms24055023/s1.
