# Supplementary material for: Usefulness and Limitations of Multiple Ligation-Dependent Probe Amplification in Antithrombin Deficiency
Source: Int J Mol Sci. 2023 Mar 6;24(5):5023. doi: 10.3390/ijms24055023 (PMC10002544; doi:10.3390/ijms24055023)
Supplement: Supplementary file 1 [file ijms-24-05023-s001.zip › ijms-2244324-supplementary.pdf]

Supplementary Table S1. Genetic, functional, and clinical characteristics of patients with antithrombin type I deficiency carrying a single nucleotide variation or a small insertion/deletion who were evaluated by MLPA. Genetic variants are related to transcript NM_000488.4.

Mutation cDNA	Protein modification	Thromb	Age 1st	Rec	AntiFXa activity	Antigen levels	Pathogenic prediction (ACMG)
c.334C>T	p.Pro112Ser	Yes	18	Yes	50%	55%	Likely Pathogenic
c.394C>T	p.Gln132Ter	Yes	22	Yes	50%	51%	Likely Pathogenic
c.839 C>A	p.Ser280Ter	Yes	25	No	50%	50%	Likely Pathogenic
c.1332_1336del	p.Arg445Serfs*18	Yes	20	Yes	50%	50%	Pathogenic
c.398A>C	p.Gln133Pro	Yes	32	Yes	60%	52%	Pathogenic
c.685C>T	p.Arg229Ter	Yes	52	No	50%	53%	Pathogenic
c.89T>A<br>c.951G>C	p.Val30Glu<br>p.Leu317Phe	Yes	ND	Yes	50%	51%	Likely Benign<br>Uncertain Significance
c.1218+1G>A	Splicing	Yes	ND	Yes	60%	50%	Likely Pathogenic
c.750del	p.Ile251Phefs*32	Yes	41	No	47%	50%	Pathogenic
c.814dup	p.Tyr272Leufs*3	Yes	57	Yes	50%	48%	Pathogenic
c.470A>G	p.Lys157Arg	Yes	25	Yes	57%	56%	Pathogenic
c.763-2A>C	Splicing	Yes	27	Yes	55%	50%	Pathogenic
c.1219-3C>A	Splicing	Yes	35	No	59%	56%	Likely Pathogenic
c.1141T>C	p.Ser381Pro	Yes	16	No	56%	66%	Uncertain Significance
c.42-2A>C	Splicing	Yes	21	Yes	17%	69%	Likely Pathogenic
c.341G>A	p.Ser114Asn	Yes	17	No	48%	49%	Pathogenic
c.1218+1G>T	Splicing	Yes	40	No	38%	44%	Likely Pathogenic
c.1246G>T<br>c.1190C>T	p.Ala416Ser<br>p.Ser397Leu	Yes	39	No	52%	52%	Pathogenic<br>Likely Pathogenic
c.1233dup	p.Ser412Glnfs*1	Yes	50	No	56%	53%	Likely Pathogenic
c.998_999insC	p.Glu334Profs*9	Yes	15	No	43%	44%	Likely Pathogenic
c.1332_1333del	p.Ile444Metfs*19	No	-	-	42%	44%	Likely Pathogenic
c.537T>G	p.Phe179Leu	No	-	-	62%	56%	Likely Pathogenic
c.243G>A	p.Trp81Ter	Yes	19	No	52%	63%	Likely Pathogenic
c.464T>C	p.Phe155Ser	Yes	20	Yes	51%	55%	Pathogenic
c.409-2A>T<br>c.1246 G>T	Splicing<br>p.Ala416Ser	Yes	31	No	35%	44%	Likely Pathogenic<br>Pathogenic
c.1033_1035del	p.Glu345del	Yes	32	Yes	59%	52%	Uncertain Significance
c.1154-2A>T	Splicing	Yes	ND	No	46%	48%	Likely Pathogenic
c.94T>C	p.Cys32Arg	Yes	25	No	45%	49%	Uncertain Significance
c.495del	p.Ala166Profs*6	Yes	18	No	44%	43%	Pathogenic
c.42-1060_1057dup	Regulatory	Yes	46	No	68%	65%	ND
c.3G>T	p.Met1Ile	Yes	16	Yes	49%	50%	Likely Pathogenic
c.1154-14G>A	Splicing	No	-	-	52%	63%	Pathogenic
c.592T>A	p.Tyr198Ans	Yes	23	No	55%	60%	Pathogenic
c.286del	p.His97Trpfs*16	Yes	40	No	56%	52%	Likely Pathogenic
c.1219-1_1248del	Splicing	Yes	29	No	58%	62%	Pathogenic
c.490C>T	p.Arg164Ter	Yes	22	No	42%	51%	Likely Pathogenic
c.1126T>C	p.Phe408Leu	Yes	19	No	42%	53%	Likely Pathogenic

c.551_553del	p.Ile218del	Yes	30	Si	42%	45%	Likely Pathogenic
c.409-1G>C	Splicing	Yes	29	No	33%	41%	Pathogenic
c.779dup	p.Lys260Lysfs*4	Yes	36	No	47%	45%	Likely Pathogenic
c.265C>A	p.Arg89Ser	Yes	57	Yes	53%	61%	Likely Pathogenic
c.749C>T	p.Thr250Ile	No	-	-	78%	82%	Likely Pathogenic
c.1171dup	p.Arg391Profs*3	No	-	-	40%	42%	Likely Pathogenic
c.1012G>T	p.Glu338Ter	Yes	ND	Yes	18%	31%	Likely Pathogenic
c.352G>C	p.Ala118Pro	Yes	ND	Yes	33%	38%	Likely Pathogenic
c.1373_1384del	p.Val458_Cys462delinsGly	No	-	-	75%	50%	Pathogenic
c.41+3A>G	Splicing	Yes	65	No	56%	52%	Uncertain Significance
c.667T>C	p.Ser223Pro	Yes	10	Yes	52%	43%	Uncertain Significance
c.344T>A	p.Ile115Asn	Yes	1	Yes	65%	50%	Likely Pathogenic
c.175G>T	p.Glu59Ter	Yes	24	No	45%	45%	Likely Pathogenic
c.666del	p.Ser223Profs*61	Yes	43	Yes	43%	48%	Likely Pathogenic
c.1115T>C	p.Leu372Pro	Yes	57	No	48%	50%	Likely Pathogenic
c.1322T>C	p.Leu441Pro	Yes	37	No	41%	38%	Pathogenic
c.1171C>T	p.Arg391Ter	Yes	40	No	40%	45%	Likely Pathogenic
c.763-1G>C	Splicing	Yes	14	Yes	37%	42%	Pathogenic
c.462_464del	p.Phe155del	Yes	31	No	25%	35%	Pathogenic
c.495delA	p.Ala166Profs*7	Yes	40	Yes	29%	38%	Pathogenic
c.1157T>C	p.Ile386Thr	No	-	-	51%	56%	Likely Pathogenic
c.580A>G	p.Ser194Gly	No	-	-	71%	78%	Likely Pathogenic
c.962 insG	p.Lys322Glufs*21	Yes	41	Yes	44%	52%	Likely Pathogenic
c.1319_1320insTT	p.Leu441Serfs*5	Yes	16	Yes	59%	60%	Pathogenic

Thromb: Thrombosis; Rec: Recurrence. ND: Not determined.

Supplementary Table S2. Location of the genetic variants identified in patients with antithrombin deficiency that could affect the MLPA probes used for SV detection in *SERPINC1*. The MLPA result obtained in each case is also shown.

Mutation cDNA	EXON	MLPA PROBE AFFECTED	POSITION	MLPA RESULT
c.41 +3 A>G	1	RPO	+7	Negative
c.302 C>G	2	LPO	-8	Negative
c.286del	2	LPO	-24	Negative
c.334 C>T	2	RPO	+25	Negative
c.335 C>T	2	RPO	+26	Negative
c.341 G>A	2	RPO	+32	Negative
c.344 T>A	2	RPO	+35	Negative
c.352 G>C	2	RPO	+43	Negative
c.592 T>A	3	RPO	+24	Negative
c.700 A>T	4	LPO	-21	Negative
c.716 T>C	4	LPO	-5	Negative
c.720 T>G	4	LPO	-1	False positive
c.722_725 delins [731_751;GAACCAG]	4	RPO	+2/+5	False positive
c.749 C>T	4	RPO	+29	Negative
c.750del	4	RPO	+30	Negative
c.1115 T>C	5	RPO	+28	Negative
c.1126 T>C	5	RPO	+39	Negative
c.1171 C>T	6	LPO	-28	Negative
c.1171dup	6	LPO	-28	Negative
c.1190 C>T	6	LPO	-8	Negative
c.1198 T>G	6	LPO	-1	False positive
c.1201 C>T	6	RPO	+3	Negative
c.1206 G >A	6	RPO	+8	Negative

c.1218+1 G >T	6	RPO	+21	Negative
c.1218+1 G >A	6	RPO	+21	Negative
c.1246 G>C	7	LPO	-14	Negative
c.1246 G>T	7	LPO	-14	Negative
c.1272_1274del	7	RPO	+13/+15	Negative
c.1277 C>T	7	RPO	+18	Negative
c.1273 C>T	7	RPO	+14	Negative
c.1274 G>A	7	RPO	+15	Negative
c.1297 A>T	7	RPO	+38	Negative
